# Adolescent oral contraceptive use and future major depressive disorder

**DOI:** 10.1192/j.eurpsy.2021.306

**Published:** 2021-08-13

**Authors:** A. De Wit, C. Anderl, E. Giltay, T. Oldehinkel, F. Chen

**Affiliations:** 1 Psychiatry, University Medical Center Groningen/University of Groningen, Groningen, Netherlands; 2 Department Of Psychology, University of British Columbia, Vancouver, Canada; 3 Department Of Psychiatry, Leiden University Medical Center, Leiden, Netherlands

**Keywords:** Oral contraceptives, adolescence, Depression

## Abstract

**Introduction:**

Previously reported associations between oral contraceptives (OCs) use and depression have been conflicting. Insight into the impact of analytical choices on the association may help to reconcile previous heterogeneous findings.

**Objectives:**

We aimed to examine the association between adolescent OC use and subsequent depression risk in early adulthood analyzing all theoretically justifiable models.

**Methods:**

Women from the prospective cohort study Tracking Adolescents’ Individual Lives Survey (TRAILS) were included in this study. All justifiable associations between adolescent OC use (ages 16-19 years) and major depressive disorder (MDD) in early adulthood (ages 20-25 years) as assessed by the Diagnostic and Statistical Manual of Mental Disorders-IV oriented Lifetime Depression Assessment Self-Report and the Composite International Diagnostic Interview were tested.

**Results:**

A total of 818 analytical models were analyzed in 534 adolescent OC users and 191 nonusers. Overall, there was a tentative association of adolescent OC use and an episode of MDD in early adulthood (median odds ratio [OR] _median_=1.41; OR_min_=1.08; OR_max_=2.18, permutation testing p-value 1 = .052, and p-value 2 = .046), which was primarily driven by the group of young women with no history of MDD (OR_median_=1.72; OR_min_=1.21; OR_max_=2.18, both permutation testing p-values = .02).

**Conclusions:**

Adolescent OC use was associated with an increased risk for experiencing an episode of MDD, but only among women with no history of MDD in adolescence. Understanding the potential side effects of OCs will help women and their doctors make informed choices when deciding among possible methods of birth control.

**Disclosure:**

No significant relationships.

